# Retraction Note: Hurdles to breakthrough in CAR T cell therapy of solid tumors

**DOI:** 10.1186/s13287-026-05046-w

**Published:** 2026-06-10

**Authors:** Faroogh Marofi, Harun Achmad, Dmitry Bokov, Walid Kamal Abdelbasset, Zeid Alsadoon, Supat Chupradit, Wanich Suksatan, Siavash Shariatzadeh, Zahra Hasanpoor, Mahboubeh Yazdanifar, Navid Shomali, Farhad Motavalli Khiavi

**Affiliations:** 1 Immunology Research Center (IRC), Tabriz University of Medical Sciences, Tabriz, Iran; 2 Department of Pediatric Dentistry, Faculty of Dentistry, Hasanuddin University, Makassar, Indonesia; 3 Institute of Pharmacy, Sechenov First Moscow State Medical University, 8 Trubetskaya St., bldg. 2, Moscow, 119991 Russian Federation; 4 Laboratory of Food Chemistry, Federal Research Center of Nutrition, Biotechnology and Food Safety, 2/14 Ustyinsky pr., Moscow, 109240 Russian Federation; 5 Department of Health and Rehabilitation Sciences, College of Applied Medical Sciences, Prince Sattam Bin Abdulaziz University, Al Kharj, Saudi Arabia; 6 Department of Physical Therapy, Kasr Al-Aini Hospital, Cairo University, Giza, Egypt; 7 Dentistry Department, College of Technical Engineering, The Islamic University, Najaf, Iraq; 8 Department of Occupational Therapy, Faculty of Associated Medical Sciences, Chiang Mai University, Chiang Mai, 50200 Thailand; 9 Faculty of Nursing, HRH Princess Chulabhorn College of Medical Science, Chulabhorn Royal Academy, Bangkok, 10210 Thailand; 10 Department of Pharmacology, School of Medicine, Shahid Beheshti University of Medical Sciences, Tehran, Iran; 11 Department of Immunology, Faculty of Medical Sciences, Tarbiat Modares University, Tehran, Iran; 12 Stem Cell Transplantation and Regenerative Medicine, Department of Pediatrics, Stanford University School of Medicine, Palo Alto, CA USA; 13 Department of Virology, Pasteur Institute of Iran, Tehran, Iran

**Retraction: Stem Cell Research & Therapy (2022) 13:140**

10.1186/s13287-022-02819-x

The Editor-in-Chief has retracted this article. An investigation by the Publisher found evidence that the authorship for this article may have been offered for sale. The Editor-in-Chief therefore no longer has confidence in the reliability of the results and conclusions of this article. Farhad Motavalli Khiavi has stated on behalf of all authors that they do not agree to this retraction.

